# Methyl 4-(4-meth­oxy­phen­yl)-2-methyl-5-oxo-1,4,5,6,7,8-hexa­hydro­quinoline-3-carboxyl­ate

**DOI:** 10.1107/S1600536810039760

**Published:** 2010-10-09

**Authors:** Xiao-Hui Yang, Yong-Hong Zhou, Meng Zhang, Xing Song

**Affiliations:** a Institute of Chemical Industry of Forest Products, Chinese Academy of Forestry, Nanjing 210042, People’s Republic of China

## Abstract

In the title compound, C_19_H_21_NO_4_, the dihydro­pyridine ring adopts a distorted screw-boat conformation. The fused cyclo­hexenone ring forms a slightly distorted envelope conformation. The dihedral angle between the mean planes of the benzene and heterocyclic rings is 86.1 (7)°. An intra­molecular C—H⋯O inter­action occurs. In the crystal, mol­ecules are linked by inter­molecular N—H⋯O hydrogen bonds, forming an infinite chain along the *c* axis.

## Related literature

For the physiological activity of 1,4-dihydro­pyridine derivatives, see: Davies *et al.* (2005 ▶); Rose & Draeger (1992 ▶); Warrior *et al.* (2005 ▶).
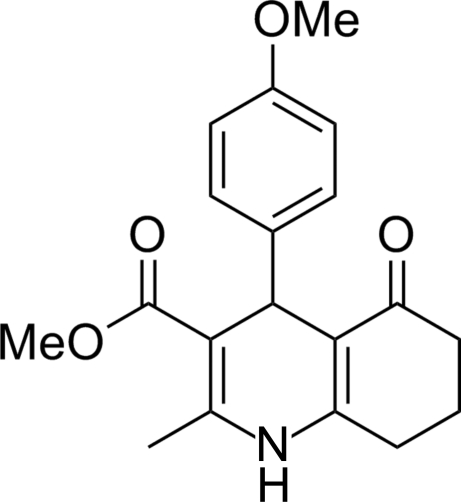


## Experimental

### 

#### Crystal data

C_19_H_21_NO_4_
*M* *_r_* = 327.37
Monoclinic, 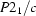

*a* = 13.628 (3) Å
*b* = 8.6300 (17) Å
*c* = 14.577 (3) Å
β = 98.39 (3)°
*V* = 1696.0 (6) Å^3^
*Z* = 4
Mo *K*α radiation
μ = 0.09 mm^−1^
*T* = 293 K
0.20 × 0.20 × 0.05 mm

#### Data collection

Enraf–Nonius CAD-4 diffractometer
Absorption correction: ψ scan (North *et al.*, 1968 ▶) *T*_min_ = 0.982, *T*_max_ = 0.996
3232 measured reflections
3040 independent reflections
1300 reflections with *I* > 2σ(*I*)
*R*_int_ = 0.078
3 standard reflections every 200 reflections  intensity decay: 1%

#### Refinement

*R*[*F* ^2^ > 2σ(*F* ^2^)] = 0.070
*wR*(*F* ^2^) = 0.128
*S* = 1.00
3040 reflections
217 parameters
1 restraint
H-atom parameters constrained
Δρ_max_ = 0.16 e Å^−3^
Δρ_min_ = −0.19 e Å^−3^

### 

Data collection: *CAD-4 EXPRESS* (Enraf–Nonius, 1994 ▶); cell refinement: *CAD-4 EXPRESS*; data reduction: *XCAD4* (Harms & Wocadlo, 1996 ▶); program(s) used to solve structure: *SHELXS97* (Sheldrick, 2008 ▶); program(s) used to refine structure: *SHELXL97* (Sheldrick, 2008 ▶); molecular graphics: *SHELXTL* (Sheldrick, 2008 ▶); software used to prepare material for publication: *SHELXTL*.

## Supplementary Material

Crystal structure: contains datablocks I, global. DOI: 10.1107/S1600536810039760/jj2057sup1.cif

Structure factors: contains datablocks I. DOI: 10.1107/S1600536810039760/jj2057Isup2.hkl

Additional supplementary materials:  crystallographic information; 3D view; checkCIF report

## Figures and Tables

**Table 1:** Hydrogen-bond geometry (Å, °)

*D*—H⋯*A*	*D*—H	H⋯*A*	*D*⋯*A*	*D*—H⋯*A*
N—H0*A*⋯O1^i^	0.86	2.05	2.884 (4)	163
C10—H10*A*⋯O3	0.96	2.08	2.818 (5)	132

Symmetry code: (i) 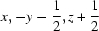.

**Fig. 1.**
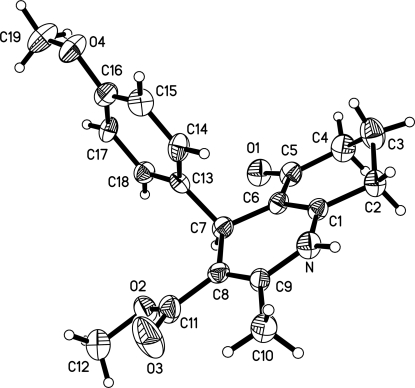
Molecular structure of the title compound, showing the atom-numbering scheme. Displacement ellipsoids are drawn at the 50% probability level and H atoms are shown as small spheres of arbitrary radii.

**Fig. 2.**
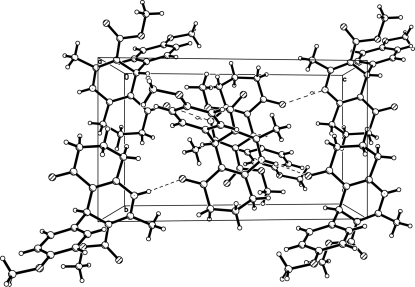
The packing of the title compound, viewed along the a axis. Dashed lines indicate N—H···O hydrogen bonds.

## supplementary crystallographic information

### Comment

The development of new methods for the synthesis of 1,4-dihydropyridine derivatives is a motive for the current study.  1,4-dihydropyridine derivatives attract interest because of their presence in numerous natural products.  In addition, they exhibit calcium modulatory properties (Rose & Draeger, 1992), antibacterial (Davies *et al.*, 2005) and fungicidal activity (Warrior *et al.*, 2005).

In the title compound the heterocyclic ring adopts a distorted screw-boat conformation (Fig. 1).  Atoms C7 and N deviate from the mean plane of C1/C6/C8/C9 by 0.177 (3)Å and 0.067 (7)Å, respectively.  The fused cyclohexene ring displays a slightly distorted envelope conformation, with atom C3 out of the plane of the atoms by -0.314 (5)°.  The dihedral angle between the mean planes of the benzene and heterocyclic rings is 86.1 (7)°. The methoxy group is nearly coplanar with the attached benzene ring with a C19/O4/C16/C17 torsion angle of -4.1 (6)°.  Crystal packing is stabilized by an intermolecular N—H···O hydrogen bond forming an infinite chain of molecules along the *c* axis (Table 1, Fig. 2).

### Experimental

A mixture of 4-methoxybenzaldehyde (2 mmol), methyl 3-oxobutanoate (4 mmol), cyclohexane-1,3-dione (2 mmol) and NH_4_CO_3_ (2 mmol) was stirred in water (2 ml) at 353 K. After completion of the reaction (TLC monitoring),the mixture was diluted with cold water (20 ml) and filtered to obtain the precipitated product which was further purified by recrystallization. Single crystals suitable for X-ray diffraction were obtained by slow evaporation of an ethanol solution.

### Refinement

Atom H0A was located in a difference map and refined isotropically. All other H atoms were positioned geometrically and treated as riding, with C—H distances in the range 0.93–0.98 Å and *U*_iso_(H) = 1.2 or 1.5 times *U*_eq_(C). In the absence of significant anomalous dispersion effects, Friedel pairs were merged.

### Crystal data

**Table:**

C_19_H_21_NO_4_	*F*(000) = 696
*M**_r_* = 327.37	*D*_x_ = 1.282 Mg m^−^^3^
Monoclinic, *P*2_1_/*c*	Mo *K*α radiation, λ = 0.71073 Å
Hall symbol: -P 2ybc	Cell parameters from 25 reflections
*a* = 13.628 (3) Å	θ = 9–12°
*b* = 8.6300 (17) Å	µ = 0.09 mm^−^^1^
*c* = 14.577 (3) Å	*T* = 293 K
β = 98.39 (3)°	Block, colourless
*V* = 1696.0 (6) Å^3^	0.20 × 0.20 × 0.05 mm
*Z* = 4	

### Data collection

**Table:**

Enraf–Nonius CAD-4 diffractometer	1300 reflections with *I* > 2σ(*I*)
Radiation source: fine-focus sealed tube	*R*_int_ = 0.078
graphite	θ_max_ = 25.3°, θ_min_ = 1.5°
ω/2θ scans	*h* = 0→16
Absorption correction: ψ scan (North *et al.*, 1968)	*k* = 0→10
*T*_min_ = 0.982, *T*_max_ = 0.996	*l* = −17→17
3232 measured reflections	3 standard reflections every 200 reflections
3040 independent reflections	intensity decay: 1%

### Refinement

**Table:**

Refinement on *F*^2^	Primary atom site location: structure-invariant direct methods
Least-squares matrix: full	Secondary atom site location: difference Fourier map
*R*[*F*^2^ > 2σ(*F*^2^)] = 0.070	Hydrogen site location: inferred from neighbouring sites
*wR*(*F*^2^) = 0.128	H-atom parameters constrained
*S* = 1.00	*w* = 1/[σ^2^(*F*_o_^2^) + (0.030*P*)^2^] where *P* = (*F*_o_^2^ + 2*F*_c_^2^)/3
3040 reflections	(Δ/σ)_max_ < 0.001
217 parameters	Δρ_max_ = 0.16 e Å^−^^3^
1 restraint	Δρ_min_ = −0.19 e Å^−^^3^

### Special details

**Table:**

Experimental. Absorption correction: semi-empirical absorption based on psi-scan (North *et al.*, 1968)
Geometry. All e.s.d.'s (except the e.s.d. in the dihedral angle between two l.s. planes) are estimated using the full covariance matrix. The cell e.s.d.'s are taken into account individually in the estimation of e.s.d.'s in distances, angles and torsion angles; correlations between e.s.d.'s in cell parameters are only used when they are defined by crystal symmetry. An approximate (isotropic) treatment of cell e.s.d.'s is used for estimating e.s.d.'s involving l.s. planes.
Refinement. Refinement of *F*^2^ against ALL reflections. The weighted *R*-factor *wR* and goodness of fit *S* are based on *F*^2^, conventional *R*-factors *R* are based on *F*, with *F* set to zero for negative *F*^2^. The threshold expression of *F*^2^ > σ(*F*^2^) is used only for calculating *R*-factors(gt) *etc*. and is not relevant to the choice of reflections for refinement. *R*-factors based on *F*^2^ are statistically about twice as large as those based on *F*, and *R*- factors based on ALL data will be even larger.

### Fractional atomic coordinates and isotropic or equivalent isotropic displacement parameters (Å^2^)

**Table:**

	*x*	*y*	*z*	*U*_iso_*/*U*_eq_	
N	0.7089 (2)	−0.1346 (4)	1.10531 (19)	0.0561 (9)	
H0A	0.7239	−0.1617	1.1624	0.067*	
O1	0.7167 (2)	−0.2573 (3)	0.79342 (17)	0.0675 (8)	
C1	0.7348 (2)	−0.2312 (4)	1.0380 (2)	0.0437 (9)	
O3	0.5828 (3)	0.3072 (4)	1.0342 (2)	0.1228 (14)	
O2	0.5564 (2)	0.2194 (3)	0.8893 (2)	0.0691 (8)	
C2	0.7719 (3)	−0.3882 (4)	1.0694 (2)	0.0579 (11)	
H2A	0.7161	−0.4545	1.0764	0.069*	
H2B	0.8130	−0.3795	1.1294	0.069*	
C3	0.8315 (3)	−0.4604 (5)	1.0013 (3)	0.0783 (14)	
H3A	0.8450	−0.5680	1.0178	0.094*	
H3B	0.8944	−0.4069	1.0040	0.094*	
O4	1.0127 (2)	0.2744 (3)	0.7949 (2)	0.0800 (9)	
C4	0.7756 (3)	−0.4514 (5)	0.9038 (3)	0.0713 (13)	
H4A	0.8194	−0.4841	0.8606	0.086*	
H4B	0.7204	−0.5234	0.8983	0.086*	
C5	0.7367 (3)	−0.2935 (5)	0.8765 (3)	0.0571 (11)	
C6	0.7201 (3)	−0.1864 (4)	0.9470 (2)	0.0444 (9)	
C7	0.6864 (3)	−0.0254 (4)	0.9202 (2)	0.0488 (10)	
H7A	0.6318	−0.0343	0.8686	0.059*	
C8	0.6458 (3)	0.0595 (4)	0.9990 (3)	0.0477 (9)	
C9	0.6599 (3)	0.0044 (4)	1.0854 (3)	0.0486 (10)	
C10	0.6220 (3)	0.0715 (4)	1.1683 (2)	0.0673 (12)	
H10A	0.5884	0.1673	1.1514	0.101*	
H10B	0.6767	0.0903	1.2165	0.101*	
H10C	0.5767	0.0000	1.1902	0.101*	
C11	0.5931 (3)	0.2038 (5)	0.9799 (3)	0.0648 (12)	
C12	0.5003 (4)	0.3605 (5)	0.8655 (3)	0.1003 (17)	
H12A	0.4785	0.3631	0.7998	0.151*	
H12B	0.5416	0.4487	0.8832	0.151*	
H12C	0.4437	0.3628	0.8977	0.151*	
C13	0.7696 (3)	0.0644 (4)	0.8845 (2)	0.0432 (9)	
C14	0.8566 (3)	0.0938 (4)	0.9442 (2)	0.0570 (11)	
H14A	0.8619	0.0646	1.0061	0.068*	
C15	0.9360 (3)	0.1667 (5)	0.9120 (3)	0.0635 (12)	
H15A	0.9937	0.1868	0.9527	0.076*	
C16	0.9296 (3)	0.2093 (5)	0.8199 (3)	0.0577 (11)	
C17	0.8445 (3)	0.1823 (4)	0.7607 (2)	0.0519 (10)	
H17A	0.8393	0.2115	0.6988	0.062*	
C18	0.7645 (3)	0.1101 (4)	0.7942 (2)	0.0500 (10)	
H18A	0.7063	0.0927	0.7537	0.060*	
C19	1.0128 (3)	0.3077 (6)	0.6991 (3)	0.0961 (17)	
H19A	1.0752	0.3531	0.6908	0.144*	
H19B	0.9602	0.3788	0.6781	0.144*	
H19C	1.0032	0.2135	0.6638	0.144*	

### Atomic displacement parameters (Å^2^)

**Table:**

	*U*^11^	*U*^22^	*U*^33^	*U*^12^	*U*^13^	*U*^23^
N	0.080 (2)	0.055 (2)	0.0358 (17)	0.0012 (18)	0.0164 (16)	−0.0055 (16)
O1	0.096 (2)	0.071 (2)	0.0380 (14)	−0.0014 (16)	0.0202 (15)	−0.0075 (15)
C1	0.047 (2)	0.039 (2)	0.046 (2)	−0.0057 (18)	0.0089 (17)	−0.0011 (18)
O3	0.212 (4)	0.069 (2)	0.091 (3)	0.048 (3)	0.033 (3)	−0.015 (2)
O2	0.076 (2)	0.0566 (19)	0.074 (2)	0.0125 (16)	0.0078 (16)	0.0084 (16)
C2	0.072 (3)	0.047 (3)	0.055 (2)	−0.002 (2)	0.008 (2)	0.002 (2)
C3	0.113 (4)	0.055 (3)	0.066 (3)	0.012 (3)	0.012 (3)	−0.005 (2)
O4	0.072 (2)	0.092 (2)	0.079 (2)	−0.0276 (18)	0.0220 (16)	0.0123 (18)
C4	0.096 (4)	0.060 (3)	0.061 (3)	0.001 (3)	0.022 (3)	−0.008 (2)
C5	0.071 (3)	0.043 (2)	0.060 (3)	−0.010 (2)	0.018 (2)	−0.013 (2)
C6	0.052 (2)	0.044 (2)	0.038 (2)	−0.0081 (19)	0.0100 (17)	−0.0026 (18)
C7	0.056 (2)	0.040 (2)	0.053 (2)	−0.0059 (19)	0.016 (2)	−0.0072 (18)
C8	0.049 (2)	0.040 (2)	0.058 (2)	−0.0060 (19)	0.0200 (19)	−0.007 (2)
C9	0.066 (3)	0.037 (2)	0.048 (2)	0.0044 (19)	0.025 (2)	0.0020 (19)
C10	0.100 (3)	0.051 (3)	0.057 (2)	0.002 (2)	0.034 (2)	−0.009 (2)
C11	0.085 (3)	0.044 (3)	0.069 (3)	−0.007 (3)	0.022 (3)	−0.003 (2)
C12	0.108 (4)	0.072 (3)	0.126 (4)	0.006 (3)	0.032 (3)	0.026 (3)
C13	0.047 (2)	0.037 (2)	0.044 (2)	0.0087 (18)	0.0037 (18)	−0.0025 (17)
C14	0.073 (3)	0.054 (3)	0.042 (2)	−0.009 (2)	0.002 (2)	0.0095 (19)
C15	0.071 (3)	0.063 (3)	0.056 (3)	−0.001 (2)	0.005 (2)	−0.002 (2)
C16	0.061 (3)	0.054 (3)	0.062 (3)	−0.010 (2)	0.021 (2)	−0.008 (2)
C17	0.068 (3)	0.047 (2)	0.042 (2)	−0.013 (2)	0.014 (2)	0.0054 (18)
C18	0.052 (2)	0.053 (3)	0.046 (2)	−0.002 (2)	0.0082 (18)	0.004 (2)
C19	0.086 (4)	0.116 (4)	0.093 (4)	−0.036 (3)	0.035 (3)	0.007 (3)

### Geometric parameters (Å, °)

**Table:**

N—C1	1.372 (4)	C7—H7A	0.9800
N—C9	1.383 (4)	C8—C9	1.334 (4)
N—H0A	0.8600	C8—C11	1.444 (5)
O1—C5	1.242 (4)	C9—C10	1.498 (4)
C1—C6	1.367 (4)	C10—H10A	0.9600
C1—C2	1.495 (4)	C10—H10B	0.9600
O3—C11	1.214 (4)	C10—H10C	0.9600
O2—C11	1.349 (4)	C12—H12A	0.9600
O2—C12	1.453 (4)	C12—H12B	0.9600
C2—C3	1.506 (5)	C12—H12C	0.9600
C2—H2A	0.9700	C13—C18	1.366 (4)
C2—H2B	0.9700	C13—C14	1.388 (4)
C3—C4	1.514 (5)	C14—C15	1.390 (5)
C3—H3A	0.9700	C14—H14A	0.9300
C3—H3B	0.9700	C15—C16	1.383 (5)
O4—C16	1.361 (4)	C15—H15A	0.9300
O4—C19	1.426 (4)	C16—C17	1.361 (5)
C4—C5	1.495 (5)	C17—C18	1.403 (4)
C4—H4A	0.9700	C17—H17A	0.9300
C4—H4B	0.9700	C18—H18A	0.9300
C5—C6	1.425 (4)	C19—H19A	0.9600
C6—C7	1.497 (4)	C19—H19B	0.9600
C7—C13	1.527 (4)	C19—H19C	0.9600
C7—C8	1.532 (4)		
			
C1—N—C9	122.9 (3)	C8—C9—C10	127.2 (4)
C1—N—H0A	118.5	N—C9—C10	112.3 (3)
C9—N—H0A	118.5	C9—C10—H10A	109.5
C6—C1—N	120.4 (3)	C9—C10—H10B	109.5
C6—C1—C2	123.3 (3)	H10A—C10—H10B	109.5
N—C1—C2	116.2 (3)	C9—C10—H10C	109.5
C11—O2—C12	115.1 (3)	H10A—C10—H10C	109.5
C1—C2—C3	111.3 (3)	H10B—C10—H10C	109.5
C1—C2—H2A	109.4	O3—C11—O2	120.2 (4)
C3—C2—H2A	109.4	O3—C11—C8	127.5 (4)
C1—C2—H2B	109.4	O2—C11—C8	112.2 (4)
C3—C2—H2B	109.4	O2—C12—H12A	109.5
H2A—C2—H2B	108.0	O2—C12—H12B	109.5
C2—C3—C4	110.6 (4)	H12A—C12—H12B	109.5
C2—C3—H3A	109.5	O2—C12—H12C	109.5
C4—C3—H3A	109.5	H12A—C12—H12C	109.5
C2—C3—H3B	109.5	H12B—C12—H12C	109.5
C4—C3—H3B	109.5	C18—C13—C14	118.0 (3)
H3A—C3—H3B	108.1	C18—C13—C7	122.6 (3)
C16—O4—C19	117.7 (3)	C14—C13—C7	119.3 (3)
C5—C4—C3	114.0 (3)	C13—C14—C15	120.4 (3)
C5—C4—H4A	108.7	C13—C14—H14A	119.8
C3—C4—H4A	108.7	C15—C14—H14A	119.8
C5—C4—H4B	108.7	C16—C15—C14	120.4 (4)
C3—C4—H4B	108.7	C16—C15—H15A	119.8
H4A—C4—H4B	107.6	C14—C15—H15A	119.8
O1—C5—C6	120.4 (4)	C17—C16—O4	124.5 (4)
O1—C5—C4	120.5 (4)	C17—C16—C15	119.8 (4)
C6—C5—C4	119.1 (3)	O4—C16—C15	115.6 (4)
C1—C6—C5	120.0 (4)	C16—C17—C18	119.2 (3)
C1—C6—C7	120.7 (3)	C16—C17—H17A	120.4
C5—C6—C7	119.2 (3)	C18—C17—H17A	120.4
C6—C7—C13	110.2 (3)	C13—C18—C17	122.1 (3)
C6—C7—C8	112.3 (3)	C13—C18—H18A	119.0
C13—C7—C8	112.3 (3)	C17—C18—H18A	119.0
C6—C7—H7A	107.2	O4—C19—H19A	109.5
C13—C7—H7A	107.2	O4—C19—H19B	109.5
C8—C7—H7A	107.2	H19A—C19—H19B	109.5
C9—C8—C11	119.3 (4)	O4—C19—H19C	109.5
C9—C8—C7	121.3 (4)	H19A—C19—H19C	109.5
C11—C8—C7	119.4 (3)	H19B—C19—H19C	109.5
C8—C9—N	120.4 (3)		
			
C9—N—C1—C6	5.7 (5)	C11—C8—C9—C10	3.5 (6)
C9—N—C1—C2	−170.1 (3)	C7—C8—C9—C10	−177.9 (3)
C6—C1—C2—C3	25.1 (5)	C1—N—C9—C8	−8.0 (5)
N—C1—C2—C3	−159.3 (3)	C1—N—C9—C10	168.2 (3)
C1—C2—C3—C4	−49.6 (5)	C12—O2—C11—O3	−3.8 (6)
C2—C3—C4—C5	49.9 (5)	C12—O2—C11—C8	178.5 (3)
C3—C4—C5—O1	158.2 (4)	C9—C8—C11—O3	22.9 (7)
C3—C4—C5—C6	−23.5 (6)	C7—C8—C11—O3	−155.7 (4)
N—C1—C6—C5	−173.0 (3)	C9—C8—C11—O2	−159.6 (4)
C2—C1—C6—C5	2.4 (5)	C7—C8—C11—O2	21.8 (5)
N—C1—C6—C7	6.9 (5)	C6—C7—C13—C18	112.6 (4)
C2—C1—C6—C7	−177.6 (3)	C8—C7—C13—C18	−121.4 (4)
O1—C5—C6—C1	174.9 (4)	C6—C7—C13—C14	−63.6 (4)
C4—C5—C6—C1	−3.4 (5)	C8—C7—C13—C14	62.4 (4)
O1—C5—C6—C7	−5.1 (5)	C18—C13—C14—C15	−0.4 (5)
C4—C5—C6—C7	176.7 (3)	C7—C13—C14—C15	175.9 (3)
C1—C6—C7—C13	110.8 (4)	C13—C14—C15—C16	−0.7 (6)
C5—C6—C7—C13	−69.3 (4)	C19—O4—C16—C17	−4.1 (6)
C1—C6—C7—C8	−15.3 (5)	C19—O4—C16—C15	174.6 (4)
C5—C6—C7—C8	164.7 (3)	C14—C15—C16—C17	1.2 (6)
C6—C7—C8—C9	13.1 (5)	C14—C15—C16—O4	−177.5 (3)
C13—C7—C8—C9	−111.8 (4)	O4—C16—C17—C18	178.0 (4)
C6—C7—C8—C11	−168.3 (3)	C15—C16—C17—C18	−0.6 (6)
C13—C7—C8—C11	66.8 (4)	C14—C13—C18—C17	1.1 (5)
C11—C8—C9—N	179.2 (3)	C7—C13—C18—C17	−175.1 (3)
C7—C8—C9—N	−2.2 (6)	C16—C17—C18—C13	−0.6 (5)

### Hydrogen-bond geometry (Å, °)

**Table:**

*D*—H···*A*	*D*—H	H···*A*	*D*···*A*	*D*—H···*A*
N—H0A···O1^i^	0.86	2.05	2.884 (4)	163
C10—H10A···O3	0.96	2.08	2.818 (5)	132

Symmetry codes: (i) *x*, −*y*−1/2, *z*+1/2.
